# Clinical Evaluation of LASEK for High Myopia Correction between the Triple-A Profile and the Zyoptix Tissue Saving Profile

**DOI:** 10.1155/2019/6936042

**Published:** 2019-04-01

**Authors:** Min Li, Yuehui Shi, Lina Sun, Lin Liu, Chen Qu, Jun Zou

**Affiliations:** Department of Ophthalmology, Shanghai Tenth People's Hospital, No. 301 Middle Yanchang Road, Shanghai 200072, China

## Abstract

**Purpose:**

To compare the effects of correcting high myopia using the MEL®90 Triple-A profile LASEK at a 500 Hz pulse rate (Triple-A group) versus the Zyoptix tissue-saving ablations of Technolas 217z laser platform at 100 Hz (TS group).

**Methods:**

This retrospective study included 50 eyes in the Triple-A group and 42 eyes in the TS group with manifest refraction spherical equivalent (MRSE) of −6 diopters (D) to −10 D. We compared uncorrected distance visual acuity, MRSE, corrected distance visual acuity, and postoperative complications at 1 month, 3 months, and 6 months.

**Results:**

At 6 months after refractive surgery, the efficacy index of Triple-A group was significantly higher than that of the TS group (1.03 ± 0.12 vs 1.00 ± 0.11, *P*=0.04). The MRSE postoperatively in the Triple-A group was significantly lower than that in the TS group (0.25 ± 0.18 vs 0.38 ± 0.23, *P* < 0.01). The safety indices in the two groups were almost the same after 6 months of surgery (1.03 ± 0.07 vs 1.04 ± 0.11, *P*=0.63). The proportion of eyes which achieved ±0.13 D was significantly higher in the Triple-A group than that in the TS group at 1 month (80% vs 59.5%, *P*=0.03), 3 months (82% vs 61.9%, *P*=0.03) and 6 months (84% vs 64.3%, *P*=0.03). The changes in refraction 6 months after surgery comparing with 1 month after surgery were 0.12 ± 0.10 D in the Triple-A group and 0.13 ± 0.08 D in the TS group (*P*=0.56). All (100%) of the patients in the Triple-A group and 50% of the patients in the TS group had a UDVA of 20/16 at 6 months after surgery (*P* < 0.01). The induced spherical aberrations and total HOAs in the Triple-A group were significantly lower than those in the TS group (0.17 ± 0.02 *μ*m vs 0.23 ± 0.02 *μ*m, *P* < 0.01; 0.20 ± 0.04 *μ*m vs 0.39 ± 0.03 *μ*m, *P* < 0.01) at 6 months after surgery. The mean reduced corneal thickness was 113.06 ± 10.5 *μ*m in the Triple-A profile group and 121.43 ± 23.46 *μ*m in the TS group (*P*=0.02). No patient in either group had haze and high intraocular pressure 6 months after surgery.

**Conclusion:**

For treatment of high-myopia patients, the Triple-A profile was more effective, predictable, and accurate than the Zyoptix tissue-saving profile. Meanwhile, the Triple-A profile had less induced spherical aberrations, total HOAs, and cornea ablation depth than the Zyoptix tissue-saving profile. Patients in the Triple-A group with 500 Hz pulse rate treatment achieved superior results. The two surgical procedures were equivalent in terms of safety and stability.

## 1. Introduction

Laser-assisted subepithelial keratomileusis (LASEK) was introduced by Camellin in 1999 [1]. It is an improved surgical approach for photorefractive keratectomy (PRK). After LASEK, patients have fewer discomfort symptoms, less nerve injury, and less dry eye, making it even more popular today [2]. Currently, spot-scanning is the most advanced profiling method. It has a faster frequency, a more uniform laser beam, and a lower central island incidence rate [3]. The Technolas 217z laser platform (Bausch and Lomb, Munich, Germany) setting on tissue-saving profile is the ideal representative of spot-scanning profiling with a spot diameter of 2 mm and a frequency of 100 Hz. It has shown itself to be effective, saving corneal tissue especially in high-myopia patients [4]. The MEL®90 (Carl Zeiss Meditec AG, Jena, Germany) when set on Triple-A profile (Advanced Ablation Algorithm) combines the advantages of both the aberration of smart ablation and tissue-saving profile because it has an ultra-small spot diameter of 0.7 mm with optional frequencies of 250 Hz and 500 Hz. Earlier, a frequency of 250 Hz was the only option when using the Triple-A profile for LASEK in order to avoid the potential risk of heat injuries associated with higher frequency. However, higher frequency settings resulted in shorter treatment time and better patient cooperation, therefore we explored the effect of using Triple-A profile with a setting frequency of 500 Hz pulse rate and compared it with the classical tissue-saving profile in high-myopia patients.

In addition to subjective quality of vision, postoperative complications and wavefront aberration are the primary factors affecting vision. The most important recognized complication after LASEK is haze, which is closely related to ablation depth [5]. Chen et al. found that the Triple-A profile with a frequency setting of 500 Hz was safe, efficient, and predictable for the correction of mild-to-moderate myopia [6]. Tandogan et al. also found that ablation frequency was not a risk factor of haze after surgery [7]. However, the effect of Triple-A profile with a frequency setting of 500 Hz for the correction of high myopia remains unknown. In the present study, we evaluated the effectiveness, safety, accuracy, stability, postoperative haze, and wavefront aberration of Triple-A profile with a frequency setting of 500 Hz and compared the results to those of the TS profile 6 months after surgery.

## 2. Materials and Methods

### 2.1. Patients

Between January 2014 and December 2015, 92 patients (92 eyes) were enrolled in this longitudinal, retrospective, observational study that was conducted at the Tenth Hospital of Shanghai, Shanghai, China, after approval from the Ethics Committee. The patients were divided into two groups: 50 eyes undergoing LASEK with Triple-A profile with a frequency setting of 500 Hz (Triple-A group) and 42 eyes undergoing LASEK with tissue-saving profile of the Technolas 217z laser platform (TS group). The inclusion criteria were age 18–40 years, stable myopia for ≥2 years, myopic spherical equivalent increment of less than −0.50 D in one year, corrected distance visual acuity of 20/25 or better, and manifest refraction spherical equivalent refraction (MRSE) of more than −6.00 D and less than −10.0 D. Patients had to discontinue wearing soft contact lenses at least 2 weeks before assessment. Exclusion criteria were a calculated postoperative residual corneal thickness of <380 *μ*m and the presence of other ocular conditions. All patients made voluntary selection of surgical methods before surgery. This study was approved by the Ethics Committee of Shanghai Tenth People's Hospital. All patients were treated in accordance with the tenets of the Declaration of Helsinki.

### 2.2. Surgical Techniques

All surgeries were performed by one surgeon. The surgical procedure was performed using 20% alcohol solution in an alcohol solution cone with 8.5 mm in diameter to create a corneal epithelial flap that was then peeled back with a crescent blade (Model 52424A; 66 Vision Tech Co., Ltd., Suzhou, China), leaving a hinge at the 12 o'clock position. Corneal stromal tissue ablations were performed in their respective groups using the Mel® 90 excimer laser at a rate of 500 Hz (Triple-A profile) with a pulse energy of 1.1 mJ or Technolas 217z laser at a rate of 100 Hz (tissue-saving profile) with a pulse energy of 400 mJ over an optical zone diameter ranging from 6.0 to 6.8 mm. The epithelial flap was then repositioned, and a bandage contact lens (ACUVE OASYS; Johnson and Johnson, New Brunswick, NJ) was inserted for 7 days. After surgery, topical steroids (fluorometholone 0.1%; Santen Pharmaceutical Co., Ltd.) were used initially every 2 hours daily and were tapered over 4 months. Topical antibiotics (levofloxacin ophthalmic solutions 0.5%; Santen Pharmaceutical Co., Ltd.) were used four times daily for 2 weeks. Artificial tears (sodium hyaluronate eye drops 0.3%; Santen Pharmaceutical Co., Ltd.) were used four times daily for more than 4 months.

### 2.3. Preoperative and Postoperative Measurements

Uncorrected distance visual acuity (UDVA), best-corrected distance visual acuity (CDVA), and MRSE were examined preoperatively and at 1, 3, and 6 months postoperatively. Intraocular pressure (Topcon KR-8900, Japan) was obtained and corrected according to the former study [8]. Corneal topography (Orbscan-II, Bausch and Lomb, American), wavefront aberration of the pupil diameter of 5 mm (Zywave, Bausch and Lomb, US), keratometer readings, central corneal thickness (Tommy SP-3000, Japan), and slit lamp examinations were performed in all eyes at each visit. The severity of postoperative subepithelial haze was graded according to a classification system devised by Dausch et al. [9] and Pallikaris et al. [10].

### 2.4. Statistical Analysis

All data were analyzed using the SPSS 19.0 software (SPSS, Inc., Chicago, IL) and shown as mean ± standard deviation. Graphs were plotted by Microsoft Excel 2010 (Microsoft Corporation, Redmond, WA). The Student's *t*-test was used to compare the data of the two groups. The one-way repeated measures ANOVA was used to compare data before and after surgery. The efficacy index and the safety index were calculated as previously described [11]. Chi-square test was used to compare the rates in accuracy and astigmatism evaluation. A *P* value of less than 0.05 was considered significantly different.

## 3. Results

Our study consisted of 92 eyes of 92 patients, 50 eyes in the Triple-A group and 42 eyes in the TS group. No eye underwent any additional treatment. The patient characteristics and preoperative MRSE are shown in Table 1. There are no significant differences in sex, age, preoperative MRSE, preoperative astigmatism, dark pupil size, and intended optical zone diameter.

### 3.1. Efficacy

**Figure 1:**
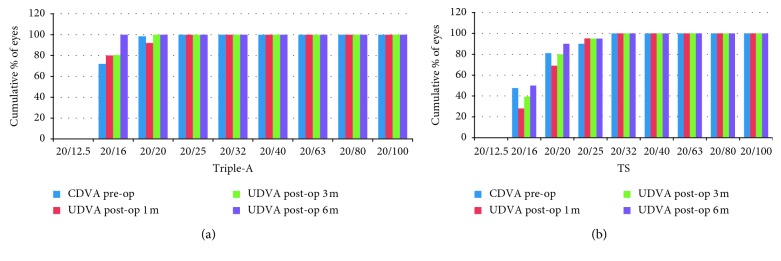
Cumulative visual acuity in the (a) Triple-A and (b) TS groups.

At 1 month after surgery, 92% of the Triple-A group patients had a UDVA of 20/20 or better and 100% patients had a UDVA of 20/25 or better, while 81% of patients in the TS group had a UDVA of 20/20 or better and 90% patients had a UDVA of 20/25 or better. All (100%) of the patients in the Triple-A group and 50% of the patients in the TS group had a UDVA of 20/16 at 6 months after surgery (Figure 1 and Table 2). The efficacy index of the Triple-A group was significantly higher than that of the TS group (*P*=0.042).

### 3.2. Safety

**Figure 2:**
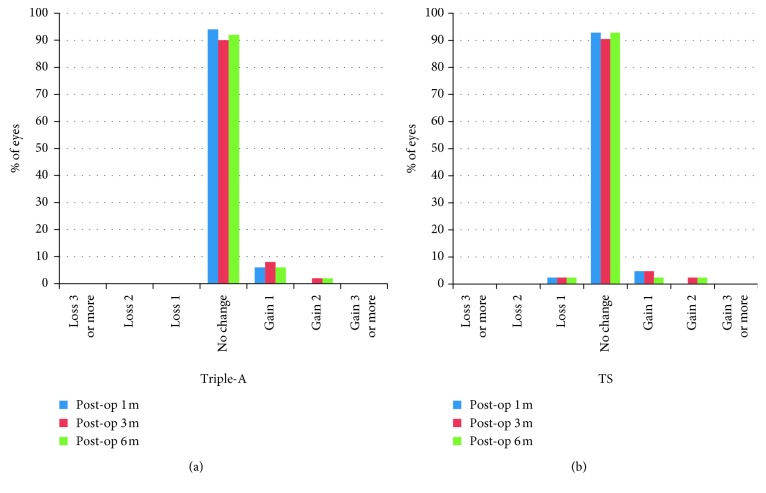
Changes in lines of CDVA in the (a) Triple-A and (b) TS groups.

After 1 month of surgery, one patient lost 1 line in the TS group, while no patient lost lines in the Triple-A group. The safety index of the Triple-A group and the TS group was almost the same (*P* > 0.05). After 6 months of surgery, four patients gained 1 line and 1 patient gained 2 lines in the Triple-A group. In the TS group, one patient lost 1 line while 2 patients gained 1 line and 1 patient gained 2 lines after 6 months of surgery (Figure 2 and Table 2). There was no significant difference in safety index between the Triple-A group and the TS group at 6 months after surgery.

### 3.3. Predictability, Accuracy, and Astigmatism

**Figure 3:**
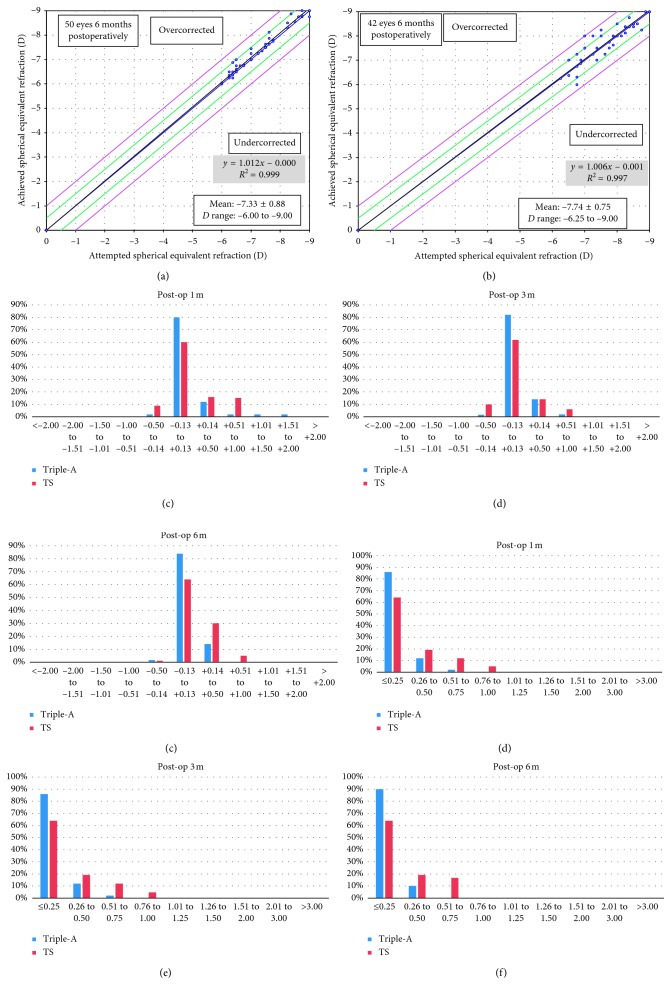
The attempted spherical equivalent refraction versus the achieved spherical equivalent refraction (a, b). The achieved spherical equivalent refraction (c–e). The astigmatism after surgery of the Triple-A and TS groups (f–h).

The proportion of eyes which achieved ±0.13 D was significantly higher in the Triple-A group than the TS group at 1 month (80% vs 59.5%, *P*=0.03) and 3 months (82% vs 61.9%, *P*=0.03) (Figures 3(c) and 3(d)). The proportion of eyes which achieved ±0.50 D of intended refraction in the Triple-A group has no significant difference compared with the TS group at 1 month (*P*=0.89) and 3 months (*P*=0.74) (Figures 3(c) and 3(d)). At 6 months after surgery, 84% of the patients in the Triple-A group achieved an MRSE of ±0.13 D, which is higher than the proportion of 64.3% in the TS group (*P*=0.03). The proportion of eyes which achieved ±0.5 D of intended refraction was higher in the Triple-A group than that in the TS group but the difference was not statistically significant (96% vs 92.9%, *P*=0.86) (Figure 3(e)). No eyes in the two groups failed to achieve ±1.00 D of intended refraction at 6 months (Figures 3(a) and 3(b)). The MRSE in the Triple-A group at 6 months after surgery was significantly lower than that in the TS group (0.25 ± 0.18 D vs 0.38 ± 0.23 D, *P*=0.003).

At 6 months after surgery, 90% of the patients in the Triple-A group achieved astigmatism of less than 0.25 D, while 64% of the patients in the TS group achieved this level. The astigmatism of 19% of the patients in the TS group was between 0.26 D and 0.50 D versus 10% of the patients in the Triple-A group (Figures 3(f)–3(h)). There was a significant difference in astigmatism between the groups (−0.33 ± 0.37 D vs −0.53 ± 0.44 D, *P*=0.019).

### 3.4. Stability

**Figure 4:**
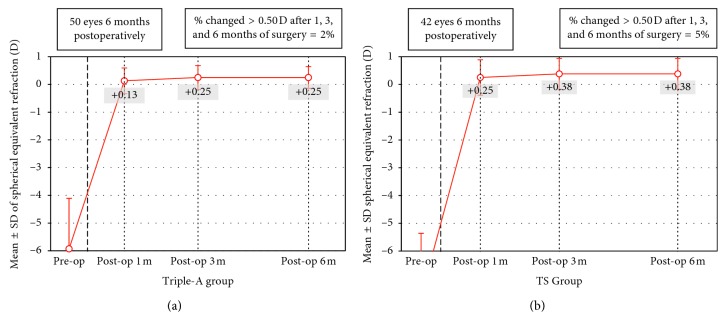
Stability of the MRSE preoperatively and after 1, 3, and 6 months in the Triple-A (a) and TS (b) groups. Two eyes in the TS group had a regression of over 0.5 D 6 months after surgery. No eye had a regression over 0.5 D in the Triple-A group.

The stabilities of refraction in the Triple-A group and the TS group are shown in Figure 4. The timeline showing the MRSE and standard deviation is represented by the error bars preoperatively, and at 1, 3, and 6 months after surgery. Two eyes in the TS group and one eye in the Triple-A group had a regression of over 0.5 D 6 months after surgery. Compared with 1 month after surgery, the changes in refraction 6 months after surgery were 0.12 ± 0.10 D in the Triple-A group and 0.13 ± 0.08 D in the TS group. The statistical analysis showed no significant difference between the groups (*P*=0.561).

### 3.5. Induced Wavefront Aberrations

**Figure 5:**
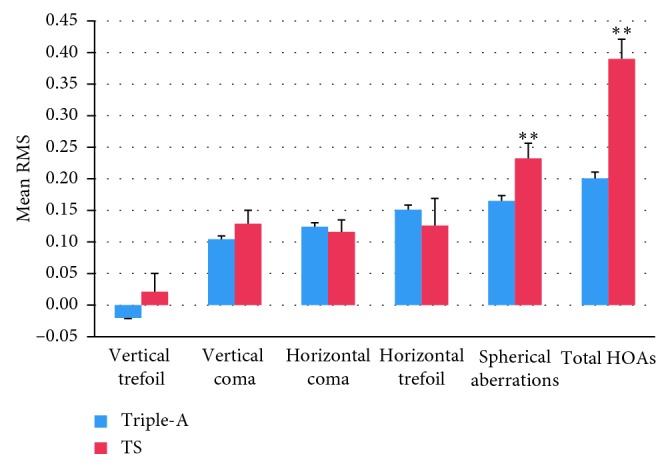
The induced wavefront aberrations of the Triple-A group and TS group 6 months after surgery.

The root mean squares (RMS) of induced wavefront aberrations 6 months after surgery in the two groups are shown in Figure 5. There was no significant difference in RMS between the Triple-A and TS groups before surgery (*P* > 0.05). However, the induced spherical aberrations and total HOAs in the Triple-A group were significantly lower than those in the TS group (0.170 ± 0.023 *μ*m vs 0.232 ± 0.024 *μ*m, *P* < 0.001; 0.201 ± 0.035 *μ*m vs 0.390 ± 0.031 *μ*m, *P* < 0.001) 6 months after surgery. There were no significant differences in terms of vertical coma, horizontal coma, vertical trefoil, and horizontal trefoil induced by surgery between the groups.

### 3.6. Ablation Depth

After 6 months of surgery, the mean reduced corneal thickness was 113.06 ± 10.5 *μ*m in the Triple-A profile group and 121.43 ± 23.46 *μ*m in the TS group. There was a significant difference in the ablation depth between the two groups (*P*=0.026).

### 3.7. Complications

After 1 month, haze (grade 1) occurred in only one eye in the TS group. Increased 0.1% fluorometholone drops use reduced haze after 1 month. There was no haze in either the Triple-A group or the TS group 6 months after surgery. There was no significant difference in the incidence rates of haze between the groups. No eyes had high intraocular pressure during the observation period.

## 4. Discussion

Ever since the profile of Zyoptix tissue-saving ablations (Technolas 217z laser platform with 100 Hz) was introduced, increasing numbers of patients with thinner corneas or high myopia have undergone this surgery. This profile can reduce the amount of ablated corneal tissue with measurement of the *K* value of the cornea [10]. The Zyoptix aspheric ablation (ASA) profile (Technolas 217z laser platform with 100 Hz) is a *Q* factor-adjusted individual ablation profile that may reduce the postoperative wavefront aberration. Both profiles have been shown to be effective, safe, and efficient [12–14]. The Triple-A profile has the advantages of both the TS profile and the ASA profile and reduces the ablation time with 1.3 second per 1 D of myopia. In our study, patients in the Triple-A (500 Hz) group showed high accuracy and effectiveness. Therefore, in effect, we demonstrated that it is possible to perform LASEK using the LASIK mode of the MEL 90 excimer laser with a 500 Hz pulse rate.

Siedlecki found that the Triple-A tissue-saving algorithm gave equally good results, whereas enhancement with the aspherically optimized profile (ASA), used in two eyes, resulted in overcorrection [15]. Dausch also reported that Triple-A was more effective than was standard aspherical surgical intervention in terms of a number of treatment outcome parameters [11] (e.g., MRSE, astigmatism, and efficacy index). The two surgical procedures were equivalent in terms of safety. In our study, the safety and stability were almost the same in the TS and Triple-A groups. However, the efficacy, accuracy, and predictability of the Triple-A group was higher than that of the TS group. Therefore, the outcomes in the Triple-A profile were superior to those in the TS profile.

One of the classical methods to prevent haze formation is using mitomycin-C (MMC) 0.01% (0.1 mg/ml) during surface ablation of the cornea [16, 17]. However, MMC carries side effects such as a refractive variation, as reported by Sy et al. [18]. In the present study, we did not use MMC in all patients during surgery. After surgery, we followed these patients closely and no haze was found in patients in the Triple-A group. Only one patient in the TS group developed haze. We immediately increased the dosage of 0.1% fluorometholone drops as reported [19] and the haze gradually faded away. Therefore, we maintain that surface keratectomy with the Triple-A profile using a frequency of 500 Hz is not a risk factor for haze formation.

The induction of HOAs can cause significant night vision problems including glare, haze, and halos. HOAs are related to the scotopic pupil size [20], the treatment optic zone [21], and the preoperative refraction [22]. Spherical aberration is usually accepted as a single aberration that is most closely affected by the degree of myopic correction performed. In our study, the pupil size, the treatment optic zone, and the preoperative SE of the two groups showed no significant difference. The spherical aberration in both groups increased after surgery. The preoperative spherical aberration and total HOAs in the Triple-A group were nearly the same as those in the TS group. However, 6 months after refractive surgery, the induced spherical aberration and total HOAs in the Triple-A group were significantly lower than those in the TS group.

This retrospective study showed that both Triple-A and TS are excellent surgical profile options for the correction of high myopia; however, the Triple-A profile with a frequency of 500 Hz pulse rate is more effective, more accurate, and more predictable despite the fact that part of the induced wavefront aberration in the Triple-A group was lower than that in the TS group. Considering that the vision and refractive status after LASEK are not stable postoperatively, long-term observations and a large sample of patients using the two profiles are needed to determine the long-term visual outcomes of LASEK.

## Figures and Tables

**Table 1:** Patient characteristics before surgery.

	Triple-A group		TS group		*P*
Sex	Female (48%)	Male (52%)	Female (47.6%)	Male (52.4%)	0.157
Age (years)	Mean: 26.48 ± 4.39	Range: 18∼35	Mean: 25.86 ± 4.65	Range: 20∼37	0.511
MRSE (D)	Mean: −7.33 ± 0.88	Range: −6.00∼−9.00	Mean: −7.66 ± 0.72	Range: −6.25 ± −9.00	0.056
Cylinder (D)	Mean: −1.03 ± 0.45	Range: 0∼−2.00	Mean: −1.15 ± 0.79	Range: 0∼−3.00	0.370
Dark pupil size	Mean: 5.91 ± 0.50	Range: 4.75∼6.75	Mean: 5.99 ± 0.46	Range: 5∼6.75	0.470
Optical zone	Mean: 6.46 ± 0.25	Range: 6.0∼6.7	Mean: 6.47 ± 0.24	Range: 6∼6.7	0.763

*Note*. Data are presented as mean ± standard deviation. D means diopter.

**Table 2:** Efficacy and safety indices in Triple-A group and TS group.

	Post 1 m		Post 3 m		Post 6 m	
	Triple-A	TS	Triple-A	TS	Triple-A	TS
Efficacy index	0.945 ± 0.100	0.887 ± 0.145^*∗*^	0.997 ± 0.134	1.021 ± 0.134	1.037 ± 0.124	0.986 ± 0.113^*∗*^
Safety index	1.058 ± 0.113	1.050 ± 0.128	1.028 ± 0.070	1.045 ± 0.088	1.032 ± 0.074	1.041 ± 0.109

*Note*. Data are presented as mean ± standard deviation. ^*∗*^*P* < 0.05 means statistically significant between the two groups. m means month.

## Data Availability

The data used to support the results of this study are included within the article.
